# Newly Acquired Fear of Falling Leads to Altered Eye Movement Patterns and Reduced Stepping Safety: A Case Study

**DOI:** 10.1371/journal.pone.0049765

**Published:** 2012-11-21

**Authors:** William R. Young, Mark A. Hollands

**Affiliations:** 1 School of Psychology, Queen’s University Belfast, Belfast, United Kingdom; 2 Research Institute for Sport and Exercise Sciences, Liverpool John Moores University, Liverpool, United Kingdom; Cardiff University, United Kingdom

## Abstract

This opportune case study describes visual and stepping behaviours of an 87 year old female (P8), both prior to, and following two falls. Before falling, when asked to walk along a path containing two stepping guides positioned before and after an obstacle, P8 generally visually fixated the first stepping guide until after foot contact inside it. However, after falling P8 consistently looked away from the stepping guide before completing the step into it in order to fixate the upcoming obstacle in her path. The timing of gaze redirection away from the target (in relation to foot contact inside it) correlated with absolute stepping error. No differences in eyesight, cognitive function, or balance were found between pre- and post-fall recordings. However, P8 did report large increases in fall-related anxiety and reduced balance confidence, supporting previously suggested links between anxiety/increased fear or falling and maladaptive visual/stepping behaviours. The results represent a novel insight into how psychological and related behavioural factors can change in older adults following a fall, and provide a possible partial rationalisation for why recent fallers are more likely to fall again in the following 12 months. These findings highlight novel possibilities for falls prevention and rehabilitation.

## Introduction

Walking safely through our cluttered world requires visual identification of hazards in our path, so that we can plan and execute appropriate stepping actions safely to avoid falling. Falls in older adults (OA) have a pronounced influence on morbidity and mortality [1], [2] which emphasises the need to identify risk-factors for falls in OA populations, and design effective tools for rehabilitation.

The detrimental effects of fear of falling (FOF) in OA has received much attention in the literature since 1982, when Murphy and Isaacs [3] described FOF as a serious disabling condition that often occurs in older adults (OA) following a fall. Increased FOF has been shown to impair performance in postural sway tasks [4] in a manner that cannot be explained by muscle weakness [5], thus predisposing OA to an increased likelihood of losing their balance and falling. FOF is also a very common condition, as between 29–92% of OA who have fallen previously will report FOF [6], [7].

Previous research has shown that older adults with FOF will adopt suboptimal eye movement patterns when walking through complex terrain [8]. More specifically, whereas OA without FOF will continue to visually fixate stepping guides until after foot contact, OA with FOF will look away from a stepping guide prematurely i.e. before completing the step in to it, in order to fixate the next hazard in the path [8]. The authors suggested that anxiety regarding future stepping hazards compels OA to fixate them earlier. This suggestion is supported by the finding that increased anxiety is associated with inducing an attentional bias, so that individuals allocate their attention towards environmental features that are perceived as being more threatening [9]. Furthermore, previous research has demonstrated a causal link between the premature transferral of gaze from a stepping guide and reduced stepping accuracy inside it, along with increased variability of foot placement [10]. This finding led the authors to suggest that maladaptive visual behaviours are likely to be associated with an increased risk of falling [10].

The current study describes a rare opportunity to compare recordings of eye movement patterns, stepping characteristics, and a range of sensory and psychological measures recorded from an 87 year old female (P8) both four weeks prior to, and following, two falls occurring within the home environment (bathroom and garden path) which each resulted in minor cuts and bruises. Designing a study such as this would be practically and ethically unfeasible as researchers cannot predict when an OA will fall for the first time. Therefore, the current results represent a particularly novel insight in to how visual/stepping characteristics can be influenced by the occurrence of a fall and associated increases in FOF.

P8 was a participant in a study reported by Young and Hollands [10]; completing two identical sessions (‘Pre-1’ and ‘Pre-2’) in the control group (i.e. there was no attempt to modify her behaviour). There were 7 days between pre-fall sessions. P8 experienced her first fall 18 days following the Pre-2 session, and fell again 5 days later. P8 repeated a further two sessions (‘Post-1’ and ‘Post-2’) 13 and 20 days following the second fall. The post-fall sessions were conducted according to the same protocol used during the pre-fall sessions, where P8 was asked to walk and accurately place her foot into a stepping guide that was followed by two further stepping hazards.

We hypothesized that anxiety or FOF directly resulting from the falls would lead to maladaptive changes in eye and stepping behaviour. Our prediction based on this hypothesis was that P8 would report increased anxiety and reduced balance confidence during the post-fall session, and would demonstrate changes in her eye and stepping behaviours characterized by earlier gaze transfer from the stepping guide resulting in reduced stepping accuracy.

## Methods

### Ethics Statement

P8 gave written and informed consent prior to the start of each experimental session. Ethical approval for both the research protocol and the consent documentation used was granted by the local ethics committee at The University of Birmingham, and was carried out in accordance with the principals laid down by the Declaration of Helsinki.

### Participant Characteristics

The battery of sensory measures taken during both pre- and post fall sessions were as follows: Peripheral cutaneous sensation under the ball of each foot was measured using a 3-point aesthesiometer. The final score from this measure represents the minimum distance between two points at which an individual can successfully discriminate their being two, as opposed to one single point. Functional balance was assessed using the Berg Balance Scale [11], where a score of 45 or below is an established criterion for identifying OA at risk of falling [12]. Binocular visual acuity was assessed in each eye using a Snellen eye chart, which provides an acuity ratio illustrating the size of a letter that can be successfully read at a given distance, where a score of <20/40 indicates a deficit in the ability to see detail. Binocular contrast sensitivity was measured in each eye using a Pelli-Robson contrast sensitivity chart (4K, Metropia Ltd, UK) at a distance of 1 meter. Scores were calculated according to the number of letters read correctly and converted to a log CS where a score of 2 represents 100% sensitivity and a score of 1 represents a disability in detecting contrasts [13]. Lower peripheral vision was measured by kinetic perimetry using a Goldmann perimeter with a V/4e target (1.75°) test spot at 320 cd/m^2^) on a backing luminance of 10 cd/m^2^. A lower visual field of >60° represents an absence of any deficit in this measure [14].

The psychological measures taken during both pre- and post-fall sessions were as follows: Falls efficacy was measured using the Falls Efficacy Scale [1]; a 10-item questionnaire where an increase in the final score represents a reduction in the participant’s confidence regarding their ability to successfully complete everyday tasks. Balance confidence was assessed using the Activities Balance Confidence scale [15]; a 16-item questionnaire where an increase in the final score represents an increase in balance confidence. State anxiety was measured using Spielberger’s State Anxiety Inventory [16]; a 20-item questionnaire asking how the participant feels at that instant, where an increase in the final score represents an increase in state anxiety. Although there is a strong association between falls-efficacy and self-reported anxiety, they are two different concepts [6], [17], [18], [19]. Therefore, we measured both in order to provide a more thorough appraisal of FOF.

Cognitive functioning was examined using the Mini Mental State Examination, where a score of >26/30 indicates no major deficit in this area [20]. Visual search and visuo-spatial memory was assessed using the Trail Making test (series B). This test requires the participant to identify sequential numbers and letters and draw lines between them on a page. The final score represents the time taken to complete the test. Results for all tests are shown in Table 1.

**Table 1:** Participant characteristics.

Measure	Pre-fall	Post-fall	% change
Weight (kg)	56.3	56.2	+0.1
Grip Strength (kg)	18.1	18.2	+0.2
Foot cutaneous sensation (3-point aesthesiometer) (cm)	1.86	1.81	−2.8
Functional balance (Berg Balance Scale) (/56)	50	50	0
Visual acuity (Snellen eye chart)	>20/40	>20/40	0
Contrast sensitivity (Pelli-robson eye chart) (Log CS)	1.5	1.5	0
Lower peripheral vision (Goldmann perimeter) (^o^)	70.7	70.1	**−0.9**
Falls Efficacy (Falls Efficacy Scale) (/100)	16	22	**+37.5**
Balance confidence (Activities Balance Confidence) (%)	80	68	**−15.0**
State anxiety (Spielberger’s Trait Anxiety Inventory) (/80)	30	37	**+23.3**
Cognitive function (Mini Mental State Examination) (/30)	30	30	0
Visual search/spatial memory (Trail making b) (s)	71	70	−1.4

Figures in bold font represent deterioration in the function/condition. In the case of state anxiety, an increase in reported anxiety was considered as deterioration.

### Walking Protocol

**Figure 1:**
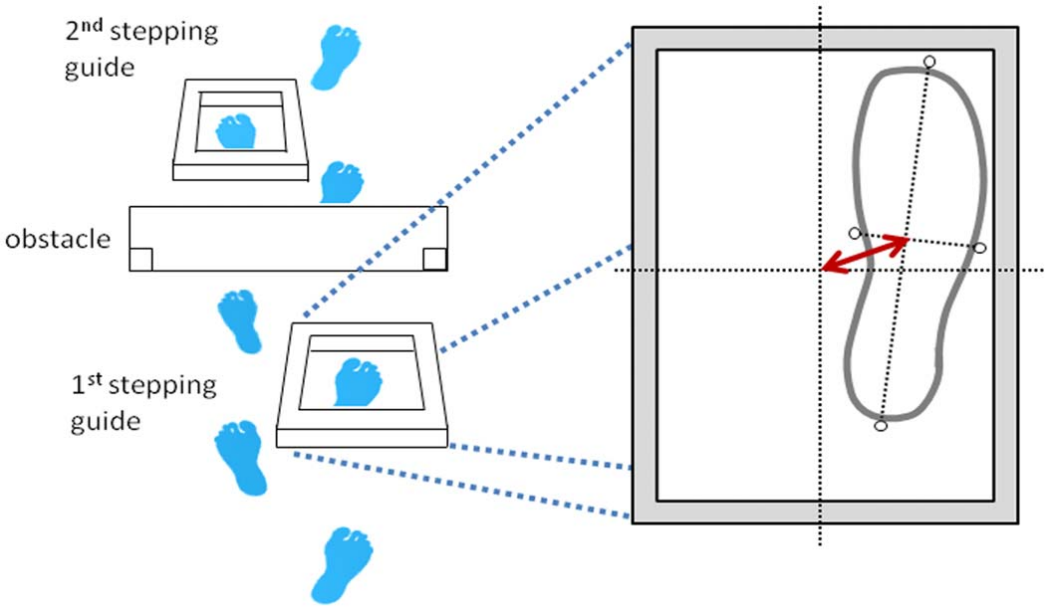
Experimental protocol. The stepping constraints are illustrated from the perspective of P8 before she started walking at the start of each trial. Typically P8 would require 5–7 steps between initiating gait at the starting position and stepping in the first stepping guide. The centres of both the foot and stepping guide were calculated as the intercept between the medio-lateral and anterior-posterior coordinates of each, as illustrated by the black dotted lines. The red arrow represents the absolute error of foot placement in the stepping guide, calculated as the distance between the central positions of the foot and stepping guide.

Within each of the two pre-fall and two post-fall sessions P8 repeated 20 trials, where she was asked to walk along a 10 m walkway and place her foot inside a stepping guide made from lightweight packaging foam, with raised edges and a boarder width of 40 mm×40 mm. Inside the stepping guide was an area of 190 mm×415 mm (width and length, respectively). The raised edges on the stepping guides were designed to encourage participants to make accurate steps into the centre of the guide by imposing a degree of postural threat. P8 was asked to place her right foot into the first stepping guide, step over the obstacle with either foot and place her left foot in the second stepping guide. In each of the stepping guides P8 was asked to place her feet “as accurately as possible into the centre of each target”. This stepping guide was followed by one stepping obstacle with a width, height, and depth of 670 mm, 210 mm and 12 mm, and a second stepping guide (Fig. 1). The obstacle was supported from the posterior side, allowing it to fall in the direction of walking should the participant make contact with it. Within each of the 20 trials in each session, both stepping guides appeared in two possible positions separated by 8 cm in both medio-lateral and anterior-posterior directions. The order of presentation was randomised. This was included in an attempt to discourage task familiarity, as the relative position of the stepping guide was altered with respect to both P8’s starting position and the following stepping hazards.

Reflective markers were placed on each corner of the initial stepping guide, and on both pairs of P8’s shoes; on the toe and heel, and on the medial and lateral sides (mid-foot markers), placed equidistantly between the toe and heel markers. The position of each marker was sampled at 120 Hz using a Vicon MX motion capture system (Oxford Metrics, England). Kinematic data were passed through a low-pass butterworth filter with a cut-off frequency of 5 Hz. The centre of the targeting foot and the centre of the stepping guide were calculated as the mid-point between the respective markers in both the medio-lateral (M-L) and anterior-posterior (A-P) planes. Absolute foot placement error was calculated at the absolute distance between the centre of the foot and the centre of the stepping guide (see Fig. 1). However, in order to assess any directional bias of foot placement error in the target we also calculated relative errors in both M-L and A-P directions, by subtracting the co-ordinate of the centre of the foot from the co-ordinate of the centre of the target in M-L and A-P directions, respectively. Toe off was determined by identifying zero crossings in the vertical velocity profile of the toe marker, and selecting that which coincided with a local minimum in toe vertical displacement.

The time of heel contact and toe-down events were determined as the maximum vertical acceleration of the heel and toe markers, respectively, identified by zero crossing in the respective jerk profiles. Foot contact times were determined as the first instance of either heel or toe contact following each step. Stance duration was defined as the elapsed time between foot contact and toe off during the single stance phase of the targeting foot prior to the step in to the stepping guide. Swing duration was calculated as the elapsed time between toe off and foot contact in the stepping guide.

Gaze behaviour was assessed using a high-speed ASL-500 head mounted gaze tracker. The horizontal and vertical components of eye-in-head angle were generated by the ASL control unit and were each sampled at 120 Hz and synchronised with the motion capture data via analog inputs. The ASL controller also produced a digital video of P8’s visual scene, superimposed with a crosshair representing the area of gaze fixation. This video was sampled at 30 Hz and used to identify the frame number in which P8 transferred her gaze from the stepping guide. This frame number was multiplied by 4 in order to select the corresponding section of the analog data. The precise timing of the saccadic eye movement away from the target was calculated by identifying an absolute local velocity peak with a threshold of 100°s^−1^ within the analogue signal representing the vertical component of eye-in-head angle. The time difference between the eye movement away from the stepping guide and foot contact inside it was then calculated for each trial.

### Data Analysis

Within a normally distributed data set, 95% of all data points lie within 2 standard deviations (2SD) of the mean. Therefore, if the difference between mean values recorded during pre- (mean of all trials from pre-1 and pre-2) and post-fall (mean of all trials from post-1 and post-2) sessions is greater than 2SD of all trials from pre-fall sessions, we can conclude that the results reflect a statistically significant difference (i.e. the results would only normally occur by chance with a probability of *p*<0.05). This analysis was carried out for each of the following variables: 1) the time of gaze transfer from the stepping guide relative to foot contact inside it, 2) absolute error with respect to the centre of the stepping guide, 3) M-L error, 4) A-P error, 5) stance duration prior to stepping in the stepping guide, 6) swing duration of the step in to the stepping guide. Pearson’s product moment correlation analysis was used to determine the extent of the relationship between the time of gaze transfer and absolute error.

For comparative group means concerning both eye and stepping behaviours in addition to scores from physiological and psychological measures see Young and Hollands [10].

## Results

### Sensory and Psychological Tests

Results from the battery of sensory and psychological tests indicated an absence of any decline greater than 1% in post-, compared to pre-fall sessions in visual acuity, contrast sensitivity, peripheral vision, peripheral cutaneous sensation in the feet, cognitive functioning, and functional balance. However, measures relating to FOF were profoundly altered following the falls, showing a 37.5% reduction in falls efficacy, 15% reduction in balance confidence, and 23.3% increase in self-reported state anxiety (Table 1). Collectively, these results suggest that from the battery of sensory and psychological tests used, only those relating to FOF showed any clear change between pre- and post-fall sessions.

### Behavioural Measures

**Figure 2:**
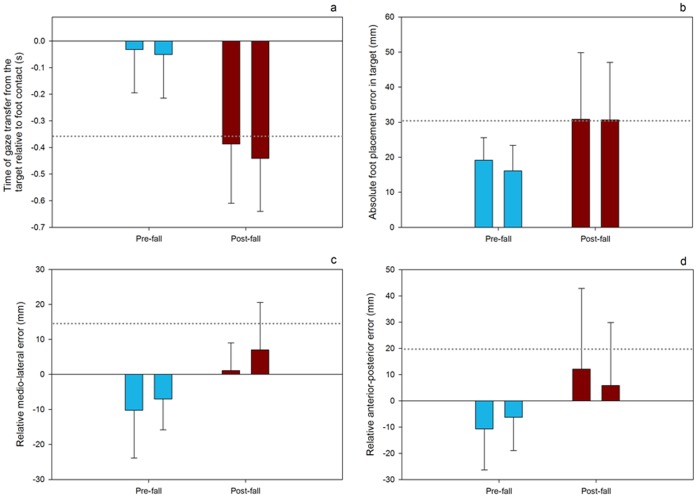
All plots represent mean values for both pre- and post-fall sessions. a) time of gaze transfer from the stepping guide, b) mean absolute error, c) M-L error (where positive values represent a lateral foot placement bias in the stepping guide), d) A-P error (where positive values represent an anterior foot placement bias in the stepping guide). Error bars represent the standard deviations of the mean for each session. Horizontal dotted lines represent 2SD above mean values from all 40 pre-fall trials.

Results for the time of gaze transfer from the stepping guide showed that P8 transferred her visual fixation from the stepping guide significantly earlier in post-fall, compared to pre-fall trials. This result is demonstrated in Figure 2a, as the magnitude of the change in the time of gaze transfer between pre- and post-fall sessions surpassed 2SD of values recorded during pre-fall sessions.

The magnitude of the change in mean absolute error between pre- and post-fall sessions did surpass 2SD of values recorded during pre-fall sessions (Fig. 2b). Furthermore, the standard deviation of absolute foot placement error increased by an average of 157% in post-fall, compared to pre-fall sessions, increasing from 6.4 mm and 7.2 mm in pre-1 and pre-2 sessions to 18.9 mm and 16.4 mm in post-1 and post-2 sessions.

In post-fall sessions P8’s foot placement in the stepping guide appears to be more lateral and anterior (Fig. 2c&d). However, these changes did not surpass 2SD of the values recorded during pre-fall sessions and therefore cannot be considered to represent a significant directional bias following the falls. The standard deviation of M-L decreased by an average of 4.7% in post-fall, compared to pre-fall sessions, decreasing from 13.7 mm and 8.8 mm in pre-1 and pre-2 sessions to 7.9 mm and 13.6 mm in post-1 and post-2 sessions. However, the standard deviation of A-P error increased by an average of 93% in post-fall, compared to pre-fall sessions, increasing from 15.6 mm and 12.6 mm in pre-1 and pre-2 sessions to 30.7 mm and 24 mm in post-1 and post-2 sessions.

There were no significant differences between pre- and post-fall sessions in measures of stance or swing duration of the step preceding the stepping guide.

**Figure 3:**
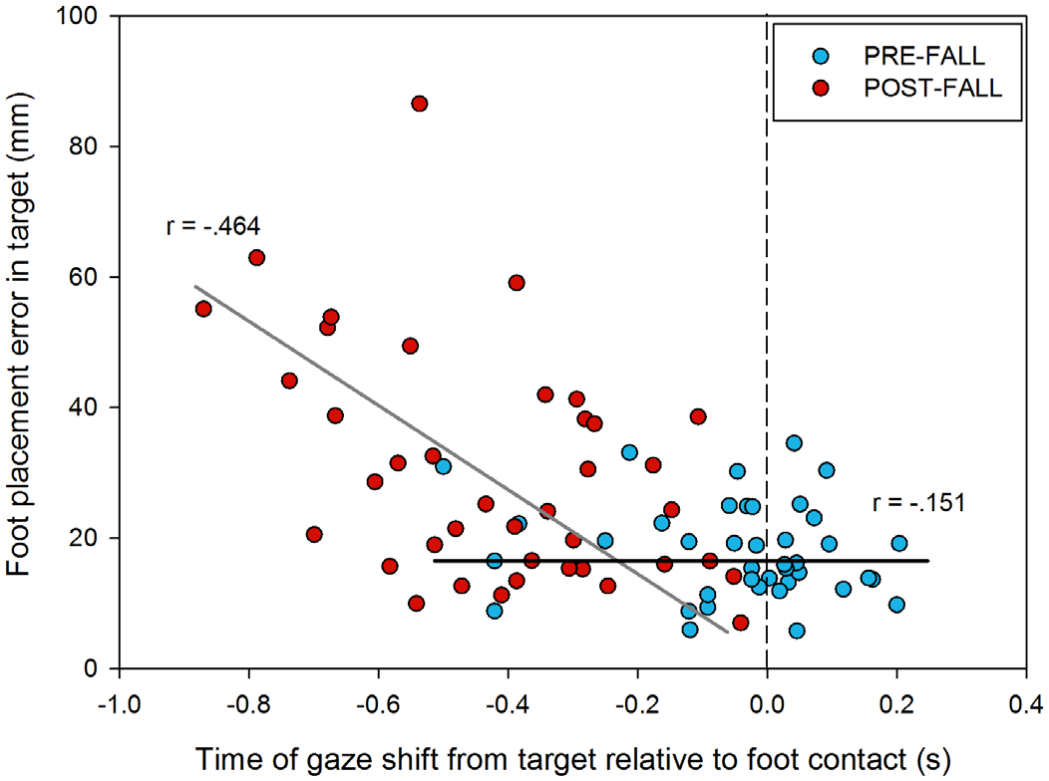
Correlation between the time of gaze transfer from the stepping guide and corresponding absolute foot placement error. Vertical dashed lines represent the time of foot contact in the stepping guide. The black and gray solid lines represent the lines of best fit for pre-fall and post-fall data, respectfully.

There was no significant correlation between eye and stepping behaviours during pre-fall trials (r_(40)_ = −.151 *p* = .351). However, there was a significant negative correlation during post-fall sessions (r_(40)_ = −.464 *p*<.005) (Fig. 3).

## Discussion

The current data show that during pre-fall sessions P8 demonstrated eye movement patterns and stepping behaviours indicative of older adults deemed to be at a low-risk of falling. However, following the falls P8 transferred her gaze away from the target significantly earlier with respect to foot contact, thus compromising her stepping accuracy; a behaviour typical of high-risk older adults [8], [21]. The similarity between mean values from both sessions within pre- and post-fall sessions for all variables illustrated in Figure 2 clearly demonstrates the repeatability of the findings. Furthermore, the results show that alterations in visual behaviour between pre- and post-fall sessions were evident even in the absence of any change in scores from tests relating to cognitive function, functional balance, or any of the other visual/physiological tests administered (Table 1).

The significant correlation between the time of gaze redirection from the stepping guide and absolute error (see Fig. 3) supports previous claims for a causal link between the two behaviours [10]. As no such correlation was found during pre-fall sessions, the data appear to suggest that the relationship between the time of gaze re-direction and stepping error is not linear. Therefore, it is pertinent for us to question the mechanisms through which visual behaviour can influence stepping accuracy.

### Visual Guidance of Foot Trajectory

Until recently it was thought that the motor plans relating to body motion during gait were fixed at foot-off, meaning that the movement of the swinging foot and centre of mass were pre-planned and ballistic in nature [22]. However, more recent studies have proposed that precision stepping actions, like reaching movements in the upper limbs, can be separated into two components; an initial ballistic transportation of the limb followed by a ‘homing in’ phase [23], [24]. Furthermore, recent findings illustrate the importance of binocular (as opposed to monocular) vision during the swing phase of gait in facilitating end-point stepping precision [25].

Chapman and Hollands [26] showed that, during a precision stepping task, foot placement accuracy is compromised in older adults when visual information regarding the stepping target is removed during the swing phase towards it [26]. This work illustrates that in older adults, visual information plays a useful role in informing alterations in foot trajectory during swing. Recent work has also shown that young and older adults (both fallers and non-fallers) can produce visually guided online corrections to stepping trajectory in response to a moving visual target [27]. However, older adults can only produce such corrections when visual information regarding the final target position is acquired prior to mid-swing (at least ∼220 ms prior to foot contact) [27]. Therefore, theoretically the relationship between the time of gaze transfer and stepping error is limited to the period preceding 220 ms prior to foot contact in the stepping guide.

Figure 3 shows that in only 5 of the 40 pre-fall trials, P8 transferred her gaze from the stepping guide over 220 ms prior to foot contact. Consequently, for the remaining 35 trials, regardless of when gaze was transferred, it would be too late to make online adjustments to improve stepping error. Furthermore, in approximately half of the pre-fall trials P8 transferred her gaze from the stepping guide after foot contact, meaning that the time of gaze transfer could not have affected foot placement accuracy at all. Collectively, these arguments provide a rationale for the lack of any significant correlation between eye and stepping behaviours in pre-fall trials. During post-fall trials P8 transferred her gaze from the stepping guide within 220 ms preceding foot contact in only 7 of the 40 trials. Therefore, in the remaining 33 trials P8 looked away from the stepping guide at a time when she could, theoretically, have still made online corrective adjustments to her foot trajectory to improve her stepping accuracy, thus providing an explanation for the significant correlation between eye and stepping behaviours in post-fall trials.

With respect to the error of P8’s foot placement in the stepping guide, we cannot conclude precisely how P8 interpreted the instructions to place her feet “as accurately as possible into the centre of each target”. For example, P8 may have considered where the ‘centre’ of her foot was and attempted to place that part of her foot on to the area where she perceived the centre of the stepping guide to be. Alternatively P8 may have attempted to place her foot in to the stepping guide so that the space around her foot was equidistant from the inside edge of the stepping guide. In the instance of the latter, we still cannot be sure whether P8 was concentrating on the placement of her whole foot, or rather just the portion of her foot visible to her (i.e. the toe). Indeed, P8’s tendency to place her foot in a more anterior position during post-fall sessions (Figure 2d) may represent a conservative strategy aimed at reducing the proximity between the part of the foot she could not see (i.e. the heel) and the posterior inside edge of the target. P8 also showed a tendency to place her foot more laterally in the target during post-fall sessions. One possible explanation for this finding could relate to previous observations that older adult fallers will often adopt a wider base of support [28]. Nevertheless, during post-fall sessions, neither M-L nor A-P error surpassed 2SD of pre-fall trials (Figure 2c&d). Therefore, any directional bias shown during post-fall sessions are unlikely to fully account for the significant increase in mean absolute error during post-fall sessions (Figure 2b).

It is curious that P8 only exhibited increased variability of foot placement in A-P, and not M-L directions during post-fall sessions. This finding is especially surprising considering that, in a similar task, older adults deemed to be at a high-risk of falling will exhibit increased M-L and A-P foot placement variability, compared to their low-risk counterparts [26]. Whilst this aspect of the current results appears to contradict the previous literature, it does lead to the suggestion that increases in mean absolute error during post-fall sessions is likely to be driven primarily by the clear increase in the variability of A-P foot placement (increase of 93%). This proposal is supported by the fact that, whilst P8 did show a systematic bias in her M-L and A-P foot placement, in both M-L and A-P planes the absolute distance of the mean foot position from the centre of the stepping guide was similar when comparing data from pre- and post-fall sessions (Figure 2c&d).

### Anxiety and Visual Behaviour

It should be considered that the test battery used was not exhaustive, and therefore we cannot unanimously state that there was no physiological change that may have influenced P8’s visual and stepping behaviours following the falls. Nevertheless, it is clear that P8 experienced a clear reduction in both balance confidence and falls-efficacy, and an increase in state anxiety following the falls, collectively indicating an increased FOF (Table 1). Based on the established relationship between heightened FOF and early gaze transfers from a stepping guide [8], it is reasonable to suggest that the corresponding changes in P8’s eye movement behaviours (and therefore decline in stepping performance) can be, at least partially, attributed to the increases in P8’s self-reported FOF, causing her to fixate upcoming stepping hazards prematurely during her approach towards them, even at the cost of jeopardizing the accuracy of concurrent stepping actions. Collectively, these within-subject changes in FOF and visual behaviour extend previous findings describing a relationship between the two [29]–[32], and illustrate the maladaptive psychological consequences that can be caused by falling.

Previous work has shown that from every 3 older adults (OA) who fall, 2 will fall again in the following 12 months [33]. But why are recent fallers more likely to fall again? Aside from any musculoskeletal injury caused from the initial fall, FOF often leads to prolonged curtailment of physical activity [34], [35], resulting in atrophy and degradation in musculoskeletal function [36]–[38]. With regards to the current results, the proximity of the experimental sessions to the falls would minimise any effects of reduced musculoskeletal function through atrophy. Furthermore, there was no reduction in P8’s grip strength following the falls; a strong predictor of overall muscle strength and physical functioning [39]. Coupled with the apparent absence of any clear decline in cognitive, balance or visual function (Table 1), our proposed relationship between increased FOF and the adoption of maladaptive eye movement patterns provide a possible, albeit partial, rationalization for why recent fallers are likely to fall again [33].

With respect to P8 it is pertinent to question what came first; the maladaptive visual strategy or the fall. Whilst we cannot reliably comment on the specific factors that predisposed P8 to fall on these occasions, we do suggest that the alterations in visual sampling will have occurred in accordance with changes in FOF [8], which presumably resulted following each of the falls [6], [7].

Importantly, the significant correlation between premature gaze transfers and increased stepping errors during post-fall sessions (Fig. 3) suggests that OA’s stepping accuracy might be improved through training eye movement patterns, such as that shown by Young and Hollands [10]. As opposed to providing training programs aimed at confronting the behavioural symptoms resulting from increased FOF, we suggest that a more effective strategy would be to provide intervention strategies aimed at rehabilitating the underlying cause; and help to improve balance confidence and reduce fall-related anxiety in OA.

In general, the nature of falls in OA will vary widely, as will the nature of each person’s recovery. Therefore, we cannot suggest that the falls experienced by P8’s are indicative of other OA’s experiences. This is a clear limitation of the current study, and an important consideration. However, our proposals for a link between FOF, eye movement patterns and stepping accuracy are based, not only on the current results, but also on the congruent relationships established in the literature [8], [10]. Therefore, the current study provides a significant contribution to this emerging area of research.

### Conclusions

After falling, P8 exhibited significant changes in her eye movement patterns, by transferring her gaze earlier from the stepping guide with respect to foot contact. P8 also showed significant reductions in stepping accuracy during post-fall trials. A significant correlation between visual and stepping behaviours during post-fall sessions support previous claims for a relationship between the two behaviours. The only changes found in the battery of sensory and psychological tests reflected a remarkable increase in P8’s FOF. Taken together, and based on previous findings from group studies, the current results support claims for a relationship between FOF and visual behaviours in OA, leading to reduced stepping accuracy.

This opportune case study provides a rare insight in to how falling can influence a range of behaviours associated with safe gait, and highlights areas that should be targeted by future falls-prevention and rehabilitation strategies.
